# Immune genes undergo more adaptive evolution than non-immune system genes in *Daphnia pulex*

**DOI:** 10.1186/1471-2148-12-63

**Published:** 2012-05-11

**Authors:** Seanna J McTaggart, Darren J Obbard, Claire Conlon, Tom J Little

**Affiliations:** 1 Institute of Evolutionary Biology; School of Biological Sciences, Ashworth Laboratories University of Edinburgh, Edinburgh, EH9 3JT, UK; 2 Centre for Immunity, Infection and Evolution; School of Biological Sciences, Ashworth Laboratories University of Edinburgh, Edinburgh, EH9 3JT, UK

**Keywords:** Host-parasite coevolution, Immune system genes, Adaptive evolution, Population genetics

## Abstract

**Background:**

Understanding which parts of the genome have been most influenced by adaptive evolution remains an unsolved puzzle. Some evidence suggests that selection has the greatest impact on regions of the genome that interact with other evolving genomes, including loci that are involved in host-parasite co-evolutionary processes. In this study, we used a population genetic approach to test this hypothesis by comparing DNA sequences of 30 putative immune system genes in the crustacean *Daphnia pulex* with 24 non-immune system genes.

**Results:**

In support of the hypothesis, results from a multilocus extension of the McDonald-Kreitman (MK) test indicate that immune system genes as a class have experienced more adaptive evolution than non-immune system genes. However, not all immune system genes show evidence of adaptive evolution. Additionally, we apply single locus MK tests and calculate population genetic parameters at all loci in order to characterize the mode of selection (directional versus balancing) in the genes that show the greatest deviation from neutral evolution.

**Conclusions:**

Our data are consistent with the hypothesis that immune system genes undergo more adaptive evolution than non-immune system genes, possibly as a result of host-parasite arms races. The results of these analyses highlight several candidate loci undergoing adaptive evolution that could be targeted in future studies.

## Background

The antagonistic interaction between host and pathogen results in adaptation and counter-adaptation in both players. The continual need for host immune systems to respond to evolving pathogens has led to the prediction that immune genes will show greater evidence of adaptive evolution than the genome average. This prediction has been supported in a recent study documenting the spatial allele frequency distributions in humans [1] and in studies comparing amino acid divergence between humans and chimpanzees [2], rodents [3], chickens [4] and between *Drosophila* species [5,6]. However, not all genes within the immune system show a strong signature of adaptive evolution, possibly reflecting the diverse functional roles that immune system genes play. For example, the *Drosophila* study of Obbard et al. [5] concluded that the global pattern was driven by a small number of genes within a limited portion of the immune system such as antiviral RNAi genes and members of the immune deficiency (IMD) pathway. In addition to the expectation that immune genes as a class will show more adaptive divergence than non-immune system genes, it has also been hypothesized that immune genes involved in the recognition of pathogens will show stronger signatures of positive selection than signal transduction molecules, which are more likely to be the subjects of purifying selection [7]. In order to test the generality of these hypotheses, the analyses of multiple immune genes need to be extended to additional species.

Here, we estimated the proportion of nucleotide sites that have been subjected to positive selection in immune versus non-immune genes in the crustacean *Daphnia pulex.* We compared a set of 30 putative immune system genes to 24 non-immune system genes. The immunity genes were initially identified by searching the *D. pulex* genome for homologous sequences to genes known to be involved in the immune system of other arthropods [8]. Coupling a phylogenetic approach such as McTaggart et al. [8] with population genetic data can provide additional support that a gene might have an immunological role, and specifically whether it is co-evolving with pathogens. Additionally, this type of approach can potentially uncover whether homologs play different roles in different taxa. Finally, population genetic theory provides the framework to identify and describe the different types of selection that might be at play in host-pathogen interactions. Our aim was to address the following questions: (1) Do immune system genes in *Daphnia* undergo greater adaptive protein evolution than non-immune system genes? (2) Which immune system genes show the strongest signature of adaptive change? (3) What type of selective pressure (i.e. directional selection or balancing selection) are the identified genes under?

## Results

We amplified 54 loci from *Daphnia pulex* (mean number of individuals = 9.1, range =2-12) and 49 loci from *D. parvula* (mean number of individuals = 2.5, range 1–4) (Table 1). We were not successful in amplifying a PCR product in all individuals at all loci. In particular, clone CC was difficult to amplify. Phylogenetic analysis indicates that CC belongs to an incipient lineage of *D. pulex*, found in western Oregon, called *D. arenata*[9], hence it was removed from all analyses, unless we failed entirely to amplify a PCR product from *D. parvula* (N = 5 loci). In the latter case, *D. arenata* samples were used as the outgroup. In total, 24 genes were classified as non-immune genes and 30 genes were classified as putative immune genes (Table 1). There was no difference in the mean sequence diversity (π) between the cyclical parthenogenic (sexual) and the obligate asexual *D. pulex* samples (paired two-sample t-test_44_ = 0.97, p = 0.33) (data not shown). Thus, all *D. pulex* samples were considered as a single group for all other tests.

**Table 1:** **Genetic diversity and Tajima's D in 54 loci in* Daphnia pulex *and an outgroup specie**s

**Gene**	**Class**^**a**^	**No. individuals(*D. pulex,*outgroup)**	**Outgroup**	**Basepairs sequenced**	**No. of alleles found**		**π**_**s**_^**b**^	**π**_**a**_^**c**^	**K**_**s**_^**d**^	**K**_**a**_^**e**^	**K**_**a**_**/K**_**s**_	**Tajima's D**			
					***D. pulex***	***D. parvula***						***D. pulex***	***p***	***D. parvula***	***p***
Actin	NI	11, 2	*D. parvula*	421	4	1	0.0082	0.0000	0.0882	0.0000	0.0000	-0.265	0.54	-	-
Caspase 1	NI	11, 4	*D. parvula*	707	9	1	0.0213	0.0000	0.0806	0.0009	0.0114	0.003	0.48	-	-
Caspase 2	NI	7, 1	*D. parvula*	655	13	2	0.0375	0.0202	0.0533	0.0236	0.4438	0.564	0.25	-	-
Caspase 3	NI	5, 1	*D. parvula*	529	8	2	0.0456	0.0109	0.1375	0.0693	0.5041	1.0170	0.14	-	-
Caspase 4	NI	11, 3	*D. parvula*	513	17	6	0.0342	0.0227	0.0923	0.0470	0.5096	0.857	0.16	1.267	0.06
Caspase 5	NI	9, 2	*D. parvula*	587	13	2	0.0354	0.0112	0.1882	0.0249	0.1323	0.201	0.36	-	-
Caspase 6	NI	8, 3	*D. parvula*	547	13	2	0.0259	0.0161	0.0804	0.0475	0.5904	0.889	0.17	0.851	0.11
Chitinase 15	NI	11, 3	*D. parvula*	877	19	5	0.0338	0.0049	0.1050	0.0215	0.2049	0.563	0.24	-0.826	0.67
Chitinase 16	NI	11, 4	*D. parvula*	931	14	6	0.0190	0.0013	0.1164	0.0174	0.1490	0.814	0.18	0.585	0.24
Chitinase 17	NI	10, 3	*D. parvula*	909	16	2	0.1514	0.0291	0.2285	0.0516	0.2260	1.808	**0.02**	-0.933	0.46
Eukaryotic translation initiation factor 2γ	NI	10, 3	*D. parvula*	1237	9	3	0.0102	0.0000	0.0350	0.0000	0.0000	0.592	0.26	-1.132	0.65
Enolase	NI	10, 4	*D. parvula*	336	3	1	0.0079	0.0000	0.1845	0.0000	0.0000	0.282	0.32	-	-
Fumerate	NI	10, 1	*D. parvula*	1103	10	2	0.0149	0.0006	0.1459	0.0039	0.0266	0.270	0.33	-	-
Glutamine-oxaloacetic transaminase	NI	10, 4	*D. parvula*	777	9	2	0.0321	0.0000	0.1368	0.0057	0.0414	1.211	0.09	1.167	0.09
Glyceraldehyde 3-phosphate dehydrogenase	NI	11, 4	*D. parvula*	701	8	1	0.0104	0.0000	0.0656	0.0038	0.0579	-0.125	0.49	-	-
Lactose dehydrogenase	NI	11, 2	*D. parvula*	840	10	1	0.0263	0.0016	0.1092	0.0024	0.0223	0.201	0.37	-	-
Mannose phosphase isomerase	NI	9, 3	*D. parvula*	751	8	2	0.0058	0.0010	0.0870	0.0010	0.0120	-0.437	0.63	-	-
Phosphoglucose isomerase	NI	11, 2	*D. parvula*	1387	17	3	0.0552	0.0025	0.1105	0.0052	0.0471	1.065	0.11	-0.780	0.53
Phosphoglycerate mutase	NI	8, 1	*D. parvula*	1184	15	2	0.0883	0.0011	0.1492	0.0056	0.0377	1.148	0.08	-	-
Scavenger 5	NI	9, 3	*D. parvula*	1440	16	5	0.0325	0.0001	0.1319	0.0069	0.0519	1.091	0.10	1.150	0.09
Scavenger 6	NI	7, 3	*D. parvula*	1119	6	5	0.0300	0.0026	0.1027	0.0113	0.1095	0.816	0.18	-0.496	0.58
Ultraspiracle	NI	10, 4	*D. parvula*	336	3	1	0.0079	0.0000	0.1845	0.0000	0.0000	0.282	0.31	-	-
*Average*				*813.0*	*11*	*3*	*0.0331*	*0.0057*	*0.1188*	*0.0159*	*0.1443*				
Caspase 8	I	2, 2	*D. parvula*	672	4	2	0.1456	0.0906	0.1994	0.1465	0.7347	2.291	**0.01**	-	-
Dicer2	I	3, 1	*D. parvula*	265	6	1	0.0731	0.0098	0.1889	0.0597	0.3162	0.029	0.37	-	-
Dorsal	I	11, 4	*D. parvula*	624	8	1	0.0242	0.0000	0.1105	0.0000	0.0000	0.003	0.43	-	-
Galectin 1	I	11, 2	*D. parvula*	715	14	4	0.0235	0.0014	0.1004	0.0142	0.1419	0.604	0.23	-0.065	0.29
Galectin 2	I	10, 4	*D. parvula*	864	8	1	0.0162	0.0005	0.2106	0.0118	0.0559	1.065	0.12	-	-
Gemini	I	11, 2	*D. parvula*	1083	15	2	0.0342	0.0002	0.0962	0.0028	0.0295	0.774	0.17	-0.755	0.45
Gram-negative binding protein 1	I	11, 3	*D. parvula*	825	13	2	0.0182	0.0027	0.1266	0.0314	0.2478	1.203	0.11	0.851	0.14
Gram-negative binding protein 10	I	11, 4	*D. parvula*	638	7	5	0.0119	0.0000	0.1150	0.0043	0.0375	0.649	0.25	-0.163	0.55
Gram-negative binding protein 11	I	11, 3	*D. parvula*	787	14	5	0.0241	0.0037	0.1419	0.0232	0.1633	0.167	0.36	0.338	0.30
Gram-negative binding protein 2	I	10, 2	*D. parvula*	748	10	3	0.0398	0.0044	0.0420	0.0099	0.2351	1.483	0.04	0.168	0.23
Gram-negative binding protein 6	I	10, 2	*D. parvula*	836	10	2	0.0229	0.0019	0.0775	0.0116	0.1495	1.366	0.07	1.633	0.27
IMD	I	10, 3	*D. parvula*	489	12	4	0.0333	0.0056	0.1504	0.0200	0.1326	-0.007	0.43	-0.933	0.43
MyD88	I	11, 3	*D. parvula*	834	8	3	0.0139	0.0016	0.1498	0.0229	0.1527	-0.243	0.52	-0.826	0.68
Nitric oxide synthase 1	I	11, 3	*D. parvula*	662	14	2	0.0229	0.0011	0.0665	0.0062	0.0926	0.056	0.44	0.851	0.14
Nitric oxide synthase 2	I	9, 2	*D. parvula*	549	13	3	0.0203	0.0047	0.1689	0.0212	0.1253	0.333	0.33	-0.612	0.26
Pelle	I	12, 2	*D. parvula*	797	7	4	0.0087	0.0000	0.0636	0.0054	0.0846	-0.523	0.66	0.592	0.38
Prophenol Oxidase	I	5, 2	*D. parvula*	1224	8	4	0.0224	0.0040	0.0235	0.0029	0.1222	-0.284	0.57	1.198	0.11
Scavenger 2	I	10, 2	*D. parvula*	1463	15	4	0.0218	0.0087	0.1355	0.0410	0.3027	-0.537	0.68	0.592	0.39
Scavenger 4	I	6, 3	*D. parvula*	841	11	2	0.0840	0.0036	0.1327	0.0108	0.0816	0.842	0.15	-	-
Stat	I	11, 3	*D. parvula*	844	8	3	0.0174	0.0000	0.5590	0.0000	0.0000	1.197	0.10	-0.144	0.54
TEP 1	I	6, 2	*D. parvula*	1067	7	3	0.0098	0.0004	0.0565	0.0102	0.1798	0.307	0.36	-0.710	0.39
TEP 2	I	4, 2	*D. parvula*	865	8	3	0.0369	0.0018	0.1556	0.0078	0.0504	0.389	0.29	-	-
TEP 3	I	9, 3	*D. parvula*	1013	14	6	0.0319	0.0009	0.1398	0.0056	0.0399	0.314	0.31	0.878	0.15
Tequila	I	3, 1	*D. parvula*	834	6	2	0.0591	0.0011	0.0581	0.0005	0.0091	0.670	0.22	-	-
Toll 1	I	9, 4	*D. parvula*	468	10	2	0.0214	0.0006	0.0938	0.0541	0.5768	1.025	0.13	-	-
Toll 2	I	10, 4	*D. parvula*	836	10	1	0.0169	0.0026	0.1106	0.0161	0.1456	0.512	0.25	-	-
Toll 3	I	9, 4	*D. parvula*	523	12	5	0.0282	0.0004	0.0887	0.0031	0.0354	-0.015	0.45	-0.431	1.39
*Average*				*791.3*	*11*	*3*	*0.0335*	*0.0056*	*0.1292*	*0.0188*	*0.1512*				
*Average over NI and I classes*				*801.1*	*10*	*3*	*0.0329*	*0.0057*	*0.1260*	*0.0182*	*0.1514*				
Scavenger 3	I	5, 1	*D. arenata*	552	8	1	0.0715	0.0057	0.0741	0.0163	0.2192	0.065	0.47	-	-
Gram-negative binding protein 7	I	9, 1	*D. arenata*	788	12	1	0.0474	0.0016	0.1150	0.0117	0.1014	-0.160	0.51	-	-
Galectin 3	I	8, 1	*D. arenata*	974	10	1	0.0232	0.0026	0.0518	0.0035	0.0678	0.326	0.31	-	-
Chitinase 1	NI	8, 1	*D. arenata*	885	13	1	0.0364	0.0072	0.0721	0.0153	0.2127	0.718	0.18	-	-
Chitinase 9	NI	6, 1	*D. arenata*	908	10	2	0.0793	0.0089	0.0748	0.0101	0.1350	0.484	1.00	-	-
*Average*				*821.4*	*10.6*	*1.2*	*0.0516*	*0.0052*	*0.0775*	*0.0114*	*0.1472*				

Table Legend: Significant P-values are indicated in bold text, (a) NI= non-immune, I: immune, (b) πs: average pairwise synonymous nucleotide diversity, (c) πa: average pairwise non-synonymous nucleotide diversity, (d) Ks: average pairwise synonymous nucleotide divergence, (e) Ka: average pairwise non-synonymous nucleotide divergence.

### Sequence diversity and divergence

There was a significant difference in the average pairwise number of synonymous substitutions per synonymous site (*K*_*s*_) between *D. pulex* and *D. arenata* compared to *D. pulex* and *D. parvula* (*t*-test, t_18_ = −3.058, p-value = 0.007) (Table 1), consistent with the much closer relationship between *D. pulex* and *D. arenata*. Since including two species in the ingroup would artificially inflate estimates of its sequence diversity, the *D. arenata* samples were excluded from the McDonald-Kreitman tests, except for 5 single-locus tests in which they comprised the outgroup. Likewise, all *D. arenata* samples were removed from the diversity and divergence calculations and multilocus analysis. Over the 49 remaining genes, the average pairwise sequence diversity between *D. pulex* and *D. parvula* at synonymous sites (π_s_) was =0.0329 and at non-synonymous sites (π_a_) was =0.0057 (Table 1), and the average pairwise sequence divergence at synonymous sites (*K*_*s*_) was 0.1260 and at non-synonymous sites (*K*_*a*_) was 0.0182 (Table 1). There is no significant difference between the average π_s_ —an estimate of 4*N*_*e*_μ —of non-immune and putative immune genes (Wilcoxon rank sum test W = 296.5, p = 1.0), or in the average π_a_ between the two gene classes (Wilcoxon rank sum test W = 311.0, p = 0.78). Similarly, there is no difference in the average divergence at either synonymous or nonsynonymous sites between the two classes of genes (*K*_*s*_: Wilcoxon rank sum test W = 314.5, p = 0.67; *K*_*a*_: Wilcoxon rank sum test W = 309.5, p = 0.81).

Two loci, *Caspase 8* and *Chitinase 17* have extremely high diversity (π_s_ =0.1456 and 0.1514, respectively) compared to the average (π_s_ =0.0329), and comparable with the average *K*_*s*_ (Table 1), which could result if two paralogous loci were sequenced instead of one, or if two highly divergent alleles were being maintained by balancing selection. BLASTn of the sequences to the *D. pulex* genome does not suggest that we had amplified two loci. To be conservative they were removed from the multilocus MK analysis. The extremely high pairwise sequence diversity (π_s_) seen for these loci also results in significantly positive Tajima’s D statistics, reflecting a high proportion of intermediate-frequency alleles (*D. pulex Caspase 8*: D = 2.29, p = 0.01; *D. pulex Chitinase 17*: D = 1.81, p = 0.02). Again, this is consistent with either the amplification of multiple loci or balancing selection. *Caspase 8* also has the highest *K*_*a*_/*K*_*s*_ ratio (0.7347) (Table 1), which is driven by an extreme excess of nonsynonymous substitutions between the species (*K*_*aCASPASE8*_ = 0.1456, *K*_*a*AVERAGE_ = 0.0182). *K*_*sCASPASE8*_ is also higher than average (=0.1994 versus 0.1260 respectively), although to a lesser extent.

### McDonald-Kreitman tests

The McDonald-Kreitman test compares the within species nucleotide diversity to between species nucleotide divergence at synonymous and non-synonymous sites [10]. Evolutionary theory predicts that if nucleotide substitutions are neutral, then the ratio of synonymous: non-synonymous changes will be equivalent within and between species. Forty of the 54 loci showed sufficient polymorphism to conduct individual McDonald-Kreitman (MK) tests (Table 2). Of these, 3 loci were significant or marginally non-significant prior to a Benjamini-Hochberg False Discovery Rate (FDR) correction (*Mannose phosphate isomerase* (*MPI*), p = 0.03; *Scavenger 3*, p = 0.04; and *Toll 1*, p = 0.05). The significance is due to an excess of nonsynonymous divergence at the *Toll 1* and *Scavenger 3* loci (indicative of positive selection), and an excess of non-synonymous polymorphism in *MPI* (indicative of weakly deleterious variants, or balancing selection). After the FDR correction for multiple tests, none of the loci show a significant deviation from the expectations under a model of neutral evolution.

**Table 2:** **McDonald-Kreitman test results at 54 loci between* Daphnia pulex *and an outgroup specie**s

**Class**^**a**^	**Gene**	***D. pulex***		***D. parvula*or*D. arenata***		**D**_**n**_^**d**^	**D**_**s**_^**e**^	**MK-test G-value (Yates correction)**	**p-value**
		**P**_**n**_^**b**^	**P**_**s**_^**c**^	**P**_**n**_	**P**_**s**_				
NI	Actin	0	4	0	0	0	8	NA	NA
NI	Caspase 1	0	12	0	0	0	8	NA	NA
NI	Caspase 2	28	18	4	9	1	0	NA	1.0000^f^
NI	Caspase 3	10	13	1	1	20	12	1.265	0.2608
NI	Caspase 4	22	10	7	7	10	4	0.134	0.7147
NI	Caspase 5	16	14	1	1	7	16	1.980	0.1594
NI	Caspase 6	27	8	1	1	12	7	0.441	0.5067
NI	Chitinase 1	12	27	0	0	5	7	0.121	0.7279
NI	Chitinase 9	18	43	0	1	2	2	0.088	0.7672
NI	Chitinase 15	5	17	11	5	6	8	0.004	0.9486
NI	Chitinase 16	3	12	7	3	9	19	0.095	0.7577
NI	Chitinase 17	48	71	3	1	13	18	0.012	0.9136
NI	Eukaryotic translation initiation factor 2γ	0	9	0	2	0	6	NA	NA
NI	Enolase	0	2	0	0	0	12	NA	NA
NI	Fumerate	2	13	0	2	3	28	0.073	0.7867
NI	Glutamine-oxaloacetic transaminase	0	15	1	1	3	17	0.130	0.7180
NI	Glyceraldehyde 3-phosphate dehydrogenase	0	4	0	0	0	9	NA	1.0000^f^
NI	Lactose dehydrogenase	4	18	0	0	0	13	NA	0.2735^f^
NI	Mannose phosphase isomerase	2	4	1	0	0	13	NA	**0.0307**^f^
NI	Phosophoglucose isomerase	9	49	1	4	2	16	0.001	0.9765
NI	Phosophoglycerate mutase	4	56	2	0	3	17	0.061	0.8044
NI	Scavenger 5	1	30	3	11	5	27	0.258	0.6114
NI	Scavenger 6	7	21	5	7	6	16	0	0.9984
NI	Ultraspiracle	0	2	0	0	0	12	NA	NA
I	Caspase 8	55	27	1	0	33	11	0.466	0.4948
I	Dicer2	5	8	0	0	10	6	1.675	0.3593
I	Dorsal	0	12	0	0	0	8	NA	NA
I	Galectin 1	2	9	0	4	5	8	1.203	0.2727
I	Galectin 2	1	6	0	0	4	18	0.109	0.7412
I	Galectin 3	9	22	0	0	1	5	0.902	0.9022
I	Gemini	2	26	1	3	2	14	0.041	0.8386
I	Gram-negative binding protein 1	5	9	0	1	18	17	0.759	0.3835
I	Gram-negative binding protein 10	0	5	1	7	2	11	0.005	0.9411
I	Gram-negative binding protein 11	8	20	2	3	8	14	0.031	0.8605
I	Gram-negative binding protein 2	5	18	5	3	1	1	0.070	0.7918
I	Gram-negative binding protein 6	5	11	2	1	5	10	0.022	0.8818
I	Gram-negative binding protein 7	5	31	0	0	5	10	1.376	0.2408
I	IMD	10	12	1	2	5	10	0.112	0.7383
I	MyD88	3	11	3	2	13	21	0.035	0.8522
I	Nitric oxide synthase 1	1	14	0	1	2	4	0.821	0.3648
I	Nitric oxide synthase 2	8	6	2	1	5	15	1.076	0.2996
I	Pelle	0	6	1	2	3	9	0.058	0.8089
I	Prophenol Oxidase	13	15	2	9	0	0	NA	NA
I	Scavenger 2	24	18	7	2	16	22	2.107	0.1467
I	Scavenger 3	7	25	0	0	5	2	4.172	**0.0411**
I	Scavenger 4	10	42	1	0	5	12	0.134	0.7146
I	Stat	0	9	0	5	0	5	NA	NA
I	TEP 1	1	7	0	2	8	11	1.979	0.1596
I	TEP 2	4	18	0	3	3	21	0.996	0.3182
I	TEP 3	2	26	1	6	3	20	0.011	0.9155
I	Tequila	2	24	0	7	0	1	NA	1.0000^f^
I	Toll 1	1	6	1	0	18	7	3.771	0.0522
I	Toll 2	5	10	0	0	8	18	0.032	0.8581
I	Toll 3	1	14	0	3	0	5	NA	1.0000^f^

Table 2 Legend: Significant P-values indicated in bold text. (a) NI= non-immune, I: immune, (b) Pn: number of nonsynonymous substitutions, (c) Ps: number of synonymous substitutions, (d) Dn: number of fixed non-synonymous differences between *D. pulex* and *D. parvula* or *D. arenata*, (e) Ds: number of fixed synonymous differences between *D. pulex* and *D. parvula* or *D. arenata*, (f) P-value based on Fisher's exact test (two-tailed), with Yate's correction.

Forty-seven out of 54 loci were included in the multilocus model (20 control genes and 27 immunity genes). Genes that used *D. arenata* as the outgroup (N = 5) were excluded from this analysis as their inclusion violates the assumption of the model that the divergence time is equal at all loci. *Caspase 8* and *Chitinase 17* were removed for the reasons described above. The results of the likelihood ratio tests amongst the four models show that both M2 and M3 are significantly better than the null model M0 (Table 3). Of these two, the Akaike information criterion strongly supports model M3, where a separate α value is estimated for each class of genes (immune versus control), over M2 (Akaike weight (*w*i) =0.96 versus 0.0 respectively) (Table 3). This is consistent with a significant permutation test between the two classes of genes (α_nonimmune_ = −0.27 (2.5 and 97.5% bootstrap intervals (BI): -1.003, 0.320), α_immune_ = 0.33 (BI: 0.029,0.607), *p* = 0.049 by permuting genes between classes).

**Table 3:** Model comparison from multilocus MK-test

**Model**	**Description**	**LnL**^**a**^	**D**^**b**^	**DF**^**c**^	**p-value**	**AICc**^**d**^	**Δι**^**e**^	***w*i**^**f**^
M0	Alpha=0 at all loci	-705.7				1548.6	6.8	0.0320
M1	Single value of alpha, estimated from the data	-705.2	1.00	1	0.3200	1551.3	9.5	0.0083
M2	Alpha varies at each locus	-637.7	136.00	47	0.0000	1680.6	138.8	0.0000
M3	Alpha values estimated for immune and for non immune system genes	-698.5	14.40	2	0.0007	1541.8	0.0	0.9600

Table 3 legend: (a) Log-likelihood, (b) Likelihood ratio test statistic (-2(ln(liklihood null model)-ln(likelihood alternative model))), (c) DF= (degrees of freedom of model 1) - (degrees of freedom of model 2), (d) AICc: Akaike information criterion corrected for finite sample sizes, (e) Di=AICci-minAICc, (f) *w*i=exp(-0.5*deltai)/sum(exp(-0.5*deltar).

The rank order between the estimate of the absolute number of adaptive nonsynonymous substitutions/nonsynonymous site (*a*) and α differed (Table 4), indicating that the level of constraint may differ among the genes. The five loci with the highest *a* values are all classed as immune system genes (*Toll 1*, *Caspase 3*, *GNBP 1*, *MyD88* and *Dicer*). However, there is not a clear split between immune and control genes; indeed 7 putative immune genes are amongst those with negative *a* values. The gene with the highest estimated *a* = 0.058 (*Toll 1*) was marginally non-significant prior to the FDR correction (p = 0.052) in the single locus MK test. The other gene that had significant MK tests prior to correcting for multiple testing (*MPI*), and was included in the multilocus MK test, was estimated to have a negative *a* value, indicating that either that some mildly deleterious alleles may be segregating at this locus, or that it was insufficiently sampled.

**Table 4:** **Estimates of** α **and* a *at 47 loci in* Daphnia pulex***

**Gene name**	**α**	***a***
Toll 1	0.91834	0.05792
Caspase 3	0.54602	0.03348
Gram -negative binding protein 1	0.81937	0.02922
MyD88	0.67028	0.01745
Dicer	0.32487	0.01632
Galectin 1	0.79554	0.01462
TEP1	0.90416	0.01148
Galectin 2	0.72113	0.00929
Chitinase 16	0.48603	0.00878
Gram-negative binding protein 11	0.39626	0.00744
Toll 2	0.38723	0.00516
Pelle	0.76734	0.00503
GOT	0.74161	0.00461
Scavenger 5	0.63212	0.00412
Gram-negative binding protein 10	0.67357	0.00367
Nitric oxide synthase 1	0.68515	0.00345
TEP3	0.53743	0.00304
Fumerate	0.51005	0.00228
Scavenger 2	0.03551	0.00119
TEP 2	0.10068	0.00061
Gemini	0.08252	0.00026
STAT	NA	0.00000
Eukaryotic translation initiation factor 2γ	NA	0.00000
Enolase	NA	0.00000
Glyceraldehyde 3-phosphate dehydrogenase	NA	0.00000
Dorsal	NA	0.00000
Caspase 1	NA	0.00000
Actin	NA	0.00000
Ultraspiracle	NA	0.00000
Gram-negative binding protein 6	-0.02889	-0.00028
Phosophoglycerate mutase	-0.22331	-0.00094
Nitric oxide synthase 2	-0.14166	-0.00227
Toll 3	NA	-0.00239
Chitinase 15	-0.15512	-0.00305
IMD	-0.19615	-0.00352
Scavenger 6	-0.41459	-0.00382
Phosophoglucose isomerase	-1.56707	-0.00402
Tequila	NA	-0.00446
Mannose phosphase isomerase	NA	-0.00526
Scavenger 4	-0.54400	-0.00528
Lactose dehydrogenase	NA	-0.00655
Caspase 4	-0.25210	-0.01110
Caspase 5	-0.57922	-0.01116
Gram negative binding protein 2	-5.40650	-0.01241
Prophenol Oxidase	NA	-0.01476
Caspase 6	-0.51729	-0.01850
Caspase 2	-19.80853	-0.04946

## Discussion

### Immune genes in *Daphnia* undergo greater adaptive evolution than non- immune genes

Consistent with the results obtained from humans [2], rodents [3], chickens [4] and fruit flies [5], our data support the hypothesis that on average immune system genes have experienced more adaptive evolution than non-immune system genes. Specifically, the most likely evolutionary model for our data fits a separate and higher value of proportion of the non-synonymous divergence due to adaptive evolution (α) for immune (=0.33) versus non-immune genes (−0.27) (Table 3). This is particularly striking since our classification of immune versus non-immune genes is likely to be conservative because, due to lack of functional information, we may have included immune genes in the non-immune gene group, which will lessen the actual the difference between the two classes. It is less likely that we have erroneously included non-immune genes in the immune class, as immune genes were only classified as such if functional information was available at homologous loci in other species. Thus, this study illustrates that the effect of host-parasite co-evolution –which is the most likely driver of adaptive change in immune system genes – is strong and consistent across a diverse taxonomic range.

As not all immune system genes exhibit evidence of adaptive evolution, our data confirm the general conclusion that immune system genes are subject to a wide range of selective pressures [3,5,7]. One explanation for this variation is that molecules interacting directly with pathogens, either in their recognition or destruction undergo more adaptive changes than molecules that do not (e.g. signal transducers). In support of this hypothesis, a study in *Drosophila* found that recognition molecules – but not signalling or effector molecules - had a significantly higher proportion of positively selected genes than the genome average [7]. Additionally, effector molecules show evidence of positive selection in frogs and termites (reviewed in [11]). In contrast, all members of the IMD pathway in *Drosophila*, including the signal transducers (except for *IMD*), showed evidence of adaptive change, while the recognition molecule that initiates this cascade did not [5]. While our dataset does not contain all members from a pathway, our results do not support the hypothesis; indeed a signal transducer (*MyD88*) in the Toll pathway is one of the five genes with the highest number of adaptive substitutions/site (*a*).

### Population genetic tests for adaptive evolution reveal likely candidate immune genes under selective pressure

In addition to testing if immune system genes as a class undergo more adaptive change than non-immune system genes, the estimates of α and *a* and/or single-locus MK tests from this study have identified several loci that may be evolving in a non-neutral manner. The first of these, *Toll 1*, encodes a transmembrane receptor that is activated by recognition proteins after fungal and bacterial invasion. *Toll 1* had the highest proportion of non-synonymous divergence due to adaptive evolution (α=0.91), as well as the highest number of adaptive substitutions per site (*a* = 0.058). Additionally, the single locus MK test was marginally non-significant because of an elevated number of nonsynonymous fixed differences between the two *Daphnia* species. Combined with the fact that this locus does not have an elevated synonymous site sequence diversity, indeed it is slightly lower than the average (π_s_ =0.02 versus 0.03), we suggest that *Toll 1* may be evolving by directional selection. In contrast, we did not detect a signature of selection in the two additional *Toll* genes included in this study (*Toll 2* and *Toll 3*). A phylogenetic analysis indicates that *Toll 1* is in a clade with *Toll 2*, while *Toll 3* is in a separate clade [8]. From this we speculate that the functionality of the *Toll 1* gene has evolved since its duplication from *Toll 2*.

Similarly, the McDonald-Kreitman test showed that the *Scavenger 3* locus had a disproportionate excess of fixed nonsynonymous substitutions between *D. pulex* and *D. arenata* (G = 4.17, P = 0.04), consistent with adaptive divergence between the two species. Scavenger receptors are responsible for binding a range of foreign ligands (including bacteria) prior to cellular internalization, and are therefore are candidate subjects of host-pathogen coevolution. At the *Scavenger 3* locus, we also observed that the proportion of silent polymorphisms in *D. pulex* was as high as silent divergence (π_s_ =0.0715 and K_s_ =0.0775 respectively), but that π_a_ was within the normal range. In contrast, Scavenger receptors in *Drosophila* have a rate of silent polymorphism that conforms to neutral expectations, but a higher rate of non-synonymous diversity compared to the genome average [12]. The observed pattern of variation in the *Daphnia Scavenger 3* locus could be due to the amplification of multiple loci, recombination between loci or balancing selection. Similarly, at the *Chitinase 17* and *Caspase 3* loci, these factors could drive the level of π_s_ to be similar to K_s_, and Tajima’s D to be positive and significant. Thus, these three loci may be interesting targets for future population genetic studies examining the effects of host-pathogen co-evolution.

Finally, the results of the single locus MK test indicates that a non-immune gene, the *MPI* locus, deviates from neutral expectations due to an excess of segregating nonsynonymous sites, which might be maintained by balancing selection. The negative value of α at this locus supports this hypothesis. However the deviation from neutral expectations could also be caused by weakly deleterious segregating alleles. The present study is unable to distinguish between these two hypotheses. Nonetheless, studies in the acorn barnacle (a crustacean) have shown that *MPI* variation is differentially selected between distinct intertidal thermal habitats [13]. The *Daphnia* sampled in this study span a large geographic distance (Table 5) and therefore may experience selective structuring among populations due to some unknown environmental gradient. Indeed, the non-synonymous sites segregating in *D. pulex* at the *MPI* locus are only found in the westernmost populations (GU and PH). Since this is the only locus where polymorphisms segregate in this manner, this pattern is not due to population subdivision. Further sampling at this locus in conjunction with an assessment of allelic fitness are needed to test if the *MPI* locus is under balancing selection in *Daphnia*, as it appears to be in the acorn barnacle.

**Table 5:** ***Daphnia *geographic origins and reproductive mode**

**Species**	**Population name**	**Population code**	**State/Province**	**Cyclical parthenogen?**
*Daphnia arenata*	Creswell Court	CC	OR	Yes
*Daphnia pulex*	Busey Woods	BW	IL	Yes
*Daphnia pulex*	Disputed Road	DI	ON	Yes
*Daphnia pulex*	Eloise Butler	EB	MN	Yes
*Daphnia pulex*	Long Point 8B	LP	ON	Yes
*Daphnia pulex*	Marion Road	MA	MI	Yes
*Daphnia pulex*	Portland Arch	PA	IN	Yes
*Daphnia pulex*	St. Guy	GU	QC	No
*Daphnia pulex*	Salisbury	SA	IN	No
*Daphnia pulex*	Fatties	FA	QC	No
*Daphnia pulex*	Pinkham Mills	PH	ME	No
*Daphnia pulex*	Linwood	LI	ON	No
*Daphnia parvula*	Action Lake	A52	OH	No
*Daphnia parvula*	Action Lake	A60	OH	No
*Daphnia parvula*	Burr Oak	C60	OH	No
*Daphnia parvula*	Burr Oak	C34	OH	No

## Conclusions

We found that, as a class, immune system genes show more adaptive evolution than non-immune system genes, supporting the hypothesis that host-parasite co-evolution is an important force in shaping genomes. As in *Drosophila*, this result appears to be driven by a few genes within the class. Our results also confirm that immune system genes experience a broad gradient of selective pressures and highlight that the targets of selection can differ among taxonomic groups. We have also identified two candidate loci under balancing selection and one under directional selection, demonstrating that the mode of action of selection in host-pathogen coevolution is variable.

## Methods

### Model system

#### Origin of samples, RNA extraction, cDNA synthesis

*Daphnia* are small aquatic crustaceans. Their primary mode of reproduction is via cyclical parthenogeneis, although some clones have lost the ability to reproduce sexually and only undergo clonal propagation. Individuals isolated from natural populations can be clonally propagated in the lab. For this experiment, a single *Daphnia pulex* clone was collected from 12 different populations from across North America. Two individual *D. parvula* clones were collected from each of 2 ponds in Ohio (Table 5). Total RNA was extracted from five *D. pulex* clones or 10 *D. parvula* clones (which are considerably smaller in size) per population using the RNeasy Midi Kit (Qiagen), following the manufacturer’s protocol, and further purified with RNAase-free DNAase (Promega). cDNA was synthesized in 20ul volumes using the Promega RT system (A3500) and subsequently diluted five-fold in ultrapure water. Two ul of this solution was used in a 20ul PCR reaction.

#### Primer design, PCR amplification, sequencing, cloning

Specific primers for all genes were designed using Primer 3 [14]. The PCR mix was as follows: 1X buffer (10 mM Tris–HCl, pH 8.3, 50 mM KCl), 1.25 mM MgCl_2_, 10uM dNTPs, 0.2uM primers, 1U Taq polymerase. PCR conditions were as follows: 94°C for 3 minutes followed by 10 cycles of 94°C for 30 seconds, 65°C for 20 seconds (−1°C/cycle), 72°C for 2 minutes. This was followed by 33 cycles of the following: 94°C for 30 seconds, 55°C for 20 seconds, 72°C for 2 minutes. A final extension step of 72°C for 3 minutes was carried out. The presence of a product of the expected size was confirmed on a 1% agarose gel.

Prior to sequencing, PCR products were cleaned by treatment with shrimp alkaline phosphatase and exonuclease I. Treated PCR products were sequenced directly in both directions using the gene specific primers in a 10 μl mix containing 1.0 μl Big Dye (Amersham), 3.0 μl 5X buffer (Amersham), 0.5 μl primer and 1–3 μl of PCR product. Sequences were aligned to the genome sequence to verify that the correct gene had been amplified.

Sequences for five of the genes (*Caspase 8*, *PPO*, *Scavenger 4*, *Toll 1* and *Eukaryotic translation initiation factor 2γ*) displayed hallmark features of indels (a ‘clean’ sequence followed by sequence with multiple peaks, which always began at the same nucleotide position, regardless of the sample). All PCR products with such a sequencing profile was cloned into the TOPO TA vector (Invitrogen), following the manufacturer’s instructions. Six of the resulting colonies were randomly chosen, added directly to a PCR mix as above, except that plasmid specific primers M13F and M13R were used. Each resulting PCR product was then sequenced as above. All sequences are available under GenBank accession numbers [JQ723152-JQ723164;JQ856341-JQ856992].

### Sequence and data analysis

Primer and vector sequences were removed from all sequences, and then aligned and edited by eye using SeqMan (DNAstar). All nucleotide positions were visually inspected to identify heterozygous peaks. We obtained pseudohaplotypes from the heterozygous sequences using the program PHASE v. 2.1 [15]. We calculated the number of synonymous and nonsynonymous polymorphisms and fixed differences using DNAsp (v5.10.01) [16]. Furthermore, we used DNAsp to calculate within species synonymous (π_s_) and nonsynonymous (π_a_) diversity, and between species synonymous (*K*_*s*_) and non-synonymous (*K*_*a*_) divergence.

Directional or balancing selection will alter allele frequencies from neutral expectations. Tajima’s *D* statistic assesses this skew in allele frequencies by taking the difference between a function of the number of segregating sites (Watterson’s θ) and the average pairwise synonymous sequence diversity (π_s_) and dividing them by their standard deviation. Since both Watterson’s θ and π_s_ are estimators of 4N_e_μ, under a null model of neutral evolution the difference between the two estimators should be zero. If *D* is negative, then there is an excess of low frequency polymorphisms, which could indicate purifying selection, population growth, or a recent selective sweep, whereas a positive value of *D* indicates that there is an excess of intermediate frequency polymorphisms, which is expected if a locus is under balancing selection, or the population is structured. We calculated Tajima’s *D* at synonymous sites for each species of *Daphnia* whenever there were at least three alleles/species available using DnaSP. Significance of this test was calculated with coalescence simulations using DnaSP.

Also using DnaSP, we conducted a McDonald-Kreitman (MK) test for each locus, which compared the observed ratios of polymorphism within a species and divergence between species to those expected under a neutral model [10]. All tests were corrected with a Benjamini-Hochberg False Discovery Rate procedure. We also conducted a multilocus MK test using the software MKtest v3.2 (modified from [17]), which estimates the proportion of non-synonymous divergence due to adaptive evolution (denoted α) for specified gene classes. We constructed the following four models: the null model (M0) assumes a single value of α=0, M1 also assumes a single value of α, but it is estimated from the data, M2 where α is estimated at each locus and finally M3 estimates a separate value of α for each of the two gene classes (immune versus non-immune).

For each model, we only varied the parameter α, and kept the remaining parameters constant as follows: the neutral diversity per site (**θ**=4N_e_μ) took a single value at all loci for each of the two *Daphnia* species, and finally, the proportion of mutations at non-synonymous sites that are under strong purifying, positive or no selection (*f*) was estimated at each locus. For each model, we calculated Akaike's Information Criterion corrected for small samples (AICc), the difference in AICc between each model and the model with the minimum AICc (Δι), and the Akaike weight (*w*i) [18]. We tested for the model with the best fit directly with the likelihood ratio test as well as *w*i. To calculate the 95% bootstrap intervals of the α estimates for M3, each gene class was bootstrapped 10 000 times and the 2.5 and 97.5% quantiles of the resulting distributions were determined. To test whether the α values estimated in M3 were significantly different from one another, we generated 10 000 permutations in which the loci were randomly assigned to one of two gene classes, and values of their respective α were estimated. We then determined if observed |α_IMMUNE_-α_NONIMMUNE_| was greater than 95% of the |α_IMMUNE_-α_NONIMMUNE_| from permuted dataset.

Ten of the loci examined are known housekeeping genes, and thus were automatically classed as non-immune system genes. Single copy genes that were known to be involved in the immune systems of other invertebrate taxa (i.e. *PelleMyD88*) and genes from multigene families that had no known function other than in immunity (i.e. Gram-negative binding proteins, TEPs, and Toll receptors) were classified as immune system genes. However, some of the genes belong to multigene families (i.e. Caspases, Chitinase and Scavenger receptors), which are known to have roles in non-immune as well as immune pathways. Thus, we used published phylogenies [8] to guide our classification of these gene family members. Any *Daphnia* gene that was in the same clade as a gene that had a empirically determined functional role in the immune system were classified as immune, while others were classified as non-immune. As functional data on many of these genes is sparse, this approach is conservative, as many putative immune genes will be classified as non-immune genes. In the case of inducible nitric oxide synthase (iNOS), where *Daphnia* has two gene copies, both were classified as putative immune system genes.

Since α is calculated as the proportion of nonsynonymous substitutions fixed by selection, it is inherently dependant on the number of nonsynonymous substitutions fixed by random genetic drift. Thus, two loci having an identical number of adaptive amino acid changes could have very different values of α if drift had fixed different numbers of nonsynonymous substitutions, i.e. if constraint also differs between the genes. Therefore, an alternate parameterisation was also used, in which “*a”* is the estimate of the absolute number of adaptive nonsynonymous substitutions/nonsynonymous site [19]. Thus, for each locus in M2 we estimated *a*, as described by the MK-Test User Guide. These values are ranked to assess which genes were experiencing the greatest evolution due to positive selection. Positive values of both α and *a* reflect the amount of positive sequence evolution, while negative numbers will result from sampling error or from the presence of mildly deleterious mutations segregating at low frequency. We attempted to correct for the effect of mildly deleterious alleles by removing all alleles that were segregating at a frequency of less than 10% prior to all analyses [20].

## Abbreviations

IMD, Immune deficiency; MK, McDonald-Kreitman; MPI, Mannose-phosphate isomerase; AICc, Akaike's Information Criterion corrected for small sample sizes.

## Competing interests

The authors declare that they have no competing interests.

## Authors’ contributions

SJM carried out the sequence alignments, analyses and wrote the manuscript. DJO guided all statistical analyses and interpretation of the data and helped draft the manuscript. CC conducted the molecular lab work and helped draft the manuscript. TJL conceived of the study and participated in its design and coordination and helped draft the manuscript. All authors read and approved the final manuscript.
